# Recent Developments in Recombinant Protein–Based Dengue Vaccines

**DOI:** 10.3389/fimmu.2018.01919

**Published:** 2018-08-23

**Authors:** Nagesh K. Tripathi, Ambuj Shrivastava

**Affiliations:** ^1^Bioprocess Scale Up Facility, Defence Research and Development Establishment, Gwalior, India; ^2^Division of Virology, Defence Research and Development Establishment, Gwalior, India

**Keywords:** dengue virus, vaccine, recombinant protein, tetravalent vaccine, chimeric vaccine

## Abstract

Recombinant proteins are gaining enormous importance these days due to their wide application as biopharmaceutical products and proven safety record. Various recombinant proteins of therapeutic and prophylactic importance have been successfully produced in microbial and higher expression host systems. Since there is no specific antiviral therapy available against dengue, the prevention by vaccination is the mainstay in reducing the disease burden. Therefore, efficacious vaccines are needed to control the spread of dengue worldwide. Dengue is an emerging viral disease caused by any of dengue virus 1–4 serotypes that affects the human population around the globe. Dengue virus is a single stranded RNA virus encoding three structural proteins (capsid protein, pre-membrane protein, and envelope protein) and seven non-structural proteins (NS1, NS2a, NS2b, NS3, NS4a, NS4b, NS5). As the only licensed dengue vaccine (Dengvaxia) is unable to confer balanced protection against all the serotypes, therefore various approaches for development of dengue vaccines including tetravalent live attenuated, inactivated, plasmid DNA, virus-vectored, virus-like particles, and recombinant subunit vaccines are being explored. These candidates are at different stages of vaccine development and have their own merits and demerits. The promising subunit vaccines are mainly based on envelope or its domain and non-structural proteins of dengue virus. These proteins have been produced in different hosts and are being investigated for development of a successful dengue vaccine. Novel immunogens have been designed employing various strategies like protein engineering and fusion of antigen with various immunostimulatory motif to work as self-adjuvant. Moreover, recombinant proteins can be formulated with novel adjuvants to enhance the immunogenicity and thus conferring better protection to the vaccinees. With the advent of newer and safer host systems, these recombinant proteins can be produced in a cost effective manner at large scale for vaccine studies. In this review, we summarize recent developments in recombinant protein based dengue vaccines that could lead to a good number of efficacious vaccine candidates for future human use and ultimately alternative dengue vaccine candidates.

## Introduction

Dengue is a mosquito-borne viral infection caused by any of 1–4 serotypes of dengue virus. These serotypes of dengue virus cross-react with each other immunologically. Dengue virus infection in human spreads by *Aedes* mosquitoes. The dengue virus is a member of the family *Flaviviridae*. The dengue cases are reported from mainly tropical and subtropical parts of the world. Recently, dengue virus has also been reported from some temperate parts of the world including France, Croatia, China, and Portugal (1). Approximately three billion people around the globe are at a risk of dengue virus infection and out of which nearly 96 million cases occur annually (2). The genetic material of dengue virus comprises of a single-stranded positive-sense RNA. There are three structural proteins (envelope, E; membrane precursor, prM; and capsid, C) and seven non-structural (NS) proteins (NS1, NS2a, NS2b, NS3, NS4a, NS4b, and NS5) that are encoded by a single open reading frame. People with primary dengue infection are at a higher risk of developing dengue hemorrhagic fever (DHF) and dengue shock syndrome (DSS) during secondary infection due to antibody-dependent enhancement (ADE) (3, 4). There is no specific antiviral treatment available for dengue infection. A dengue vaccine (Dengvaxia) has been recently licensed for human use; however, it is unable to provide balanced protection against all the serotypes. Therefore, there is a need to develop dengue vaccine which should provide balanced immune response.

The development of an efficacious vaccine against all serotypes of dengue virus is possible. However, many challenges like tetravalent formulations, new correlates of protection, and long-term protection are associated with the dengue vaccine development (5). The first licensed vaccine for dengue in the world is a live attenuated tetravalent vaccine (Dengvaxia) developed by Sanofi Pasteur (6, 7). However, Dengvaxia is not intended for a population with low sero-prevalence so as to minimize the potential risk of severe dengue in a vaccinated seronegative individual (8). In a recent clinical efficacy trial, this vaccine led to increased risk of ADE in the seronegative vaccinated individuals (8). The success of dengue vaccines in future mainly depends on understanding the complex interactions of antigens of dengue virus in human hosts (9). Apart from recently licensed Dengue vaccine (Dengvaxia), various other dengue vaccine candidates based on purified inactivated virus, live attenuated virus, recombinant subunit vaccine, DNA vaccine, virus-vectored vaccine, and virus- like particles (VLPs) vaccine approach are in different stages of vaccine development (5, 10, 11). These vaccines primarily elicit an immune response to the whole dengue virus or the envelope proteins.

Various recombinant proteins produced using microbes and higher organisms are used as a therapeutic agents and vaccine candidates (12–14). The expression host system mostly used for recombinant dengue virus proteins include bacteria (15), yeast (16), mammalian cells (17), insect cells (18), transgenic animals (19), and transgenic plants (20). The recombinant proteins are mainly produced with the help of genetic engineering techniques. *E. coli*, a prokaryotic organism, is most commonly employed for heterologous expression of proteins as this organism offers fast growth rate with high product yield. Expression of recombinant proteins in *E. coli* is easy and economical; however, its lacks post-translational modifications (PTM) that are critical for various biological activity (13, 21). Yeast systems (*Saccharomyces cerevisiae* and *Pichia pastoris*) are utilized due to their ability to express recombinant proteins with PTM. This yeast system leads to proper folding of protein together with glycosylation. However, this glycosylation pattern is not similar to that of human in some cases (22, 23). Recombinant proteins produced using mammalian cells result in good protein quality and also glycosylation pattern (12, 24). Insect cells and transgenic plants are also used for expression of recombinant dengue virus proteins. Recombinant dengue virus proteins expressed using the above hosts are evaluated in preclinical trials and some of them were also evaluated in phase 1 clinical trials. In this review, we have discussed on recent developments in recombinant dengue virus proteins for vaccine application. Further, we have also discussed about current status of dengue vaccine development using different approaches.

## Dengue virus proteins

Dengue virus encodes three structural proteins: C protein, M protein and E protein, and seven non-structural proteins. The structural proteins are required for assembly of the virus. Dengue virus C protein is a highly basic protein of about 11 kDa size. It helps in the assembly of nucleocapsid with the help of RNA interaction (25). C protein of enveloped viruses are emerging as promising targets for a new generation of antiviral agents. A recent high-throughput screening identified a small molecule (ST-148) that potently inhibits replication of all four dengue virus serotypes *in vitro* by targeting the C protein. This makes C an attractive target for dengue virus inhibition (26). Membrane associated protein is an M glycoprotein of about 26 kDa. It helps E protein to form a mature virus particle. The M protein can help in differentially identifying the immune responses against various flaviviruses (27). M protein oligomerizes and forms the structure of virus particle (28). E protein, a major constituent of virus particle of about 55 kDa, is a surface protein and is responsible for the attachment and fusion of the virus to the host cell membrane. It contains two N-linked glycosylation sites at Asn67 and Asn153. The E ectodomain, also termed soluble E (sE) protein comprises of three distinct regions namely domain I (structural domain), domain II (dimers) and domain III (binding domain). DI forms a β-sheet occupying a central position in the mature monomer. DII forms an elongated finger-like structure having two distal loops; the most distal one serves as the internal fusion loop during fusion to host cell membranes. Additionally, DII provides the surface where the main interactions for E dimerization occur (29, 30). A linker (11 aa) connecting DI to DIII is crucial for proper E folding (31). The type-specific neutralizing epitopes of dengue virus 2 have been mapped on the lateral ridge of domain I (DI), the dimer interface, lateral ridge, and fusion loop of DII, and the lateral ridge, C-C′ loop, and A strand of DIII. Five monoclonal antibodies (MAbs) against these epitopes conferred passive protection in BALB/c mice challenged with dengue virus 2 (32). The E domain III (EDIII) is the important immunogen, which elicits neutralizing antibody titer and also responsible for cellular receptor binding. The utility of EDIII protein as a vaccine candidate has also been well studied for dengue vaccine development (33, 34).

The non-structural proteins include NS1, NS2a, NS2b, NS3, NS4a, NS4b, and NS5. These proteins help in replication of virus and other functions in the host cells. The role of dengue virus NS proteins was recently well reviewed with respect to pathophysiology, host immune evasion and other physiological changes in the microenvironment of the cells during dengue infection (35). The NS1 glycoprotein of 46 kDa is cleaved from E protein by host signal peptidase. NS1 is responsible for viral RNA replication. During infection of the host cells, the NS1 is generally secreted from infected cells. This secreted NS1 is a hexamer and accumulates in serum in high amounts. Thus, it can be targeted by immunological methods for the diagnosis of acute dengue infection (36–41). NS1 protein contains epitopes associated with major histocompatibility complex (MHC I) and MHC II molecules that are targets for T cells and thus can be targeted as a vaccine candidate (42). Antiviral drugs targeting the NS1 protein acts by interfering with the N-glycosylation of the protein, which is required for its biological activity (43). NS2a is a 22 kDa hydrophobic protein which helps in synthesis of viral RNA and further in assembly/maturation of the virus particle (44). NS2a inhibits IFN-β promoter driven transcription. Further, a single-amino-acid mutation in NS2a can reduce this inhibitory activity substantially. These findings identify NS2a as a target for attenuation in the development of live flavivirus vaccines (45). It is also reported that the N-terminal half of NS2a protein of dengue virus 2 is implicated in causing cytopathic effect (CPE) due to virus, whereas the C-terminal portion takes part in assembly of virus particle and subsequent secretion from the cell. This fact can be exploited for development of vaccines and drugs against dengue infection (46). NS2b is a 14 kDa membrane-associated protein. NS2b together with NS3 forms a stable complex. This complex act as a cofactor for the NS2b-NS3 protease. NS2b cleaves NS2a/NS2b and NS2b/NS3 complexes (47). Dengue virus 2 NS2b oligomerizes to form a pore-like structures in various lipid environments, thus altering the permeability of cell membranes (48). NS3, a 69 kDa protein comprises of two domains, an N-terminal protease (170 aa) and a C-terminus helicase or RNA triphosphatase (440 aa). The NS2b/NS3 protease is the first dengue protein target used for drug development (49). The 3D structures of NS2b, NS3 of dengue virus 2 have paved the ways for the discovery of new potent antiviral compounds effective against dengue virus 1-4 serotypes (50). The NS3 is the most immunodominant target for cellular responses (51). The NS4a is a 16 kDa size and involved in replication cycle, membrane rearrangements and their regulation (52). NS4b, a 27 kDa protein is crucial for replication of virus and virus-host interactions. It was proved experimentally that a potential antiviral can be designed by blocking the NS4a and NS4b interactions (53). NS5 is the largest and most conserved dengue protein of 100 kDa. NS5 encodes RNA dependent RNA polymerase which is mainly responsible for RNA replication (54, 55). Due to RNA-dependent RNA polymerase (RdRp) activity, NS5 can be targeted for the development of new antiviral drugs (56). Since NS5 gene contains epitopes for CD8+ T cells in dengue virus infections, therefore this protein can be included as a part of the vaccine candidate (51). In a recent review, the structure and functions of NS5, a protein responsible for the replication and capping of viral RNA that represents a promising drug target was well described (57).

## Dengue vaccine candidates based on live attenuated or purified inactivated virus

Various dengue vaccine candidates are presently undergoing advanced stage of clinical trials based on purified inactivated virus or live attenuated virus approach (5, 10, 11). The current status of dengue vaccine development based on live attenuated or inactivated virus is given in Table 1. These vaccine candidates have their own merits and demerits based on different strategies. Till now, Sanofi Pasteur CYD-TDV has been licensed under the trade name of Dengvaxia (58). Other important vaccine candidates in the advanced clinical trials are TV003/TV005 (NIH), TDV (Takeda/Inviragen), and TDEN (WRAIR/GSK) (59–62). Recently licensed dengue vaccine (CYD-TDV) uses prM/E of dengue virus 1-4 on YF-17D backbone. TV003 and TV005 (identical to TV003 except the dosing level of the dengue virus 2 component) are based on wild-type strains with genetic mutations to attenuate the virus (64). TDV (formerly DENVax) is also a live recombinant vaccine, which contains a whole attenuated dengue virus 2 PDK53 and chimeric dengue virus 1, 3, 4 on the dengue virus 2 PDK53 backbone. TDEN-LAV (WRAIR/GSK), a new live-attenuated dengue virus candidate prepared from re-derived PDK vaccine strains, except that each strain has three additional passages in fetal rhesus lung cells (FRhL) (65). Studies on another inactivated dengue virus monovalent and tetravalent vaccines (TDEN-PIV vaccine-WRAIR/GSK/FIOCRUZ) with adjuvant were carried out in mice and non-human primate (NHP). Further, TDEN-PIV induced strong neutralizing antibodies with T cell responses thus conferring protection (63, 66).

**Table 1:** Current status of dengue vaccine candidates based on live attenuated or purified inactivated virus.

**Vaccine candidate and developer**	**Approach and type of vaccine**	**Status**	**References**
Dengvaxia® (CYD-TDV); Sanofi Pasteur	YF-17D backbone with prM and E genes of dengue virus 1-4 (Live attenuated vaccine)	Licensed	(58)
TetraVax-DV-TV003/TV005; NIAID/NIH	Full length dengue virus 1/2/3/4 lacking 30 nt in 3′UTR (Live attenuated vaccine)	Phase III	(59, 60)
DENVax/TAK003/TDV; Takeda/Inviragen	Dengue virus 2 PDK53 backbone with dengue virus 1,3 and 4 prM and E gene chimera (Live attenuated vaccine)	Phase III	(61)
TDEN; WRAIR/GSK	Dengue virus 1/2/3/4 PDK virus (Live attenuated vaccine)	Phase II	(62)
TDEN PIV; WRAIR/GSK/Fiocruz	Formalin inactivated dengue virus 1/2/3/4 (Purified inactivated vaccine)	Phase I	(63)

## DNA vaccine candidates for dengue virus

The plasmid DNA vaccine uses plasmids, as vector for expressing antigens of dengue virus *in vivo*. Thus, they permit efficient delivery of genes into the host cells (10, 17, 67, 68). The list of DNA based dengue vaccine candidates is given in Table 2. The frontrunner in the DNA vaccine candidate is TVDV/D1ME100 [Naval Medical Research Center (NMRC), USA], which has completed phase I trial. The D1ME100 vaccine is a monovalent DNA plasmid vaccine expressing the prM/E genes of dengue virus 1. D1ME100 was evaluated at the NMRC and found to be safe (68, 69). During phase I trials of D1ME100 though IFN-γ T cell response was obtained yet a very low percentage of participants elicited neutralizing antibody response. Subsequently, the NMRC is pursuing another strategy where the tetravalent dengue DNA vaccine based on similar approach is being adjuvanted with Vaxfectin®. This adjuvanted vaccine resulted in enhanced titer of neutralizing antibodies against dengue virus 1, 3, 4 when compared to DNA vaccine alone (TVDV/D1ME100) in NHPs. This tetravalent adjuvanted DNA vaccine has completed phase I study (10, 64, 69).

**Table 2:** List of DNA, virus-vectored, VLPs, and recombinant protein based dengue vaccine candidates.

**Vaccine candidates and type**	**Approach**	**Status/Animal model**	**References**
D1ME100/ TVDV (Monovalent)-NMRC, USA (DNA vaccine)	Dengue virus 1 prM and E protein expressed under control of human cytomegalovirus promoter of VR1012 vector	Phase I	(69)
pcTPANS1 (DNA vaccine)	Dengue virus 2 NS1 protein-TPA transfected in BHK-21 cells	Preclinical/Mice	(70)
αDEC-NS1 (DNA vaccine)	Dengue virus 2 NS1 protein transfected in HEK cells	Preclinical/Mice	(71)
DENV3 prM/E (DNA vaccine)	Dengue virus 3 prM and E protein transfected in HeLa cells	Preclinical/Mice	(72)
DENV4 prM/E (DNA vaccine)	Dengue virus 4 prM and E protein generated using mammalian expression plasmid pCI	Preclinical/Mice	(73)
DENV1-4 E (DNA vaccine)	Dengue virus 1-4 E ectodomain	Preclinical/Mice	(17)
DEN EDIII-CH3 (DNA vaccine)	Dengue virus EDIII protein linked to CH3 domain of IgG H chain	Preclinical/Mice	(74)
DENV2 EDIIII scaffold/DNA (DNA vaccine)	Protein particle (60 copies of EDIII presented on a multimeric scaffold of *Geobacillus stearothermophilus* E2 proteins) and a DNA expression plasmid (EDIII portion cloned into *E. coli*)	Preclinical/NHPs	(75)
MV-DENV1-4 EDIII (Tetravalent) (Virus-vectored vaccine)	Dengue virus 1-4 EDIII protein and dengue virus 1 ectoM expressed from live attenuated measles virus vector	Preclinical/NHPs	(76)
DENV1-4 E85-VEE (Tetravalent) (Virus-vectored vaccine)	Dengue virus 1-4 soluble E dimer (E85) protein expressed from single-cycle VEE virus vector	Preclinical/NHPs	(77)
DENV2 DIII-S; hybrid DIII-S (Virus-vectored vaccine)	Dengue virus 2 DIII protein and Hepatitis B surface (S) antigen (HBsAg); Hybrid antigen displaying DIII-S on an unmodified HBsAg scaffold expressed from two MV vectors	Preclinical/Mice	(78)
MV-DENV1-4 EDIII (Tetravalent) (Virus-vectored vaccine)	Dengue virus 1-4 EDIII protein expressed from MV vector	Preclinical/Mice	(79)
rMVA/Sg-E (Virus-vectored vaccine)	Dengue virus 3 E protein with signal peptide expressed from MVA vector	Preclinical/Mice	(80)
DENV2 E85-RRV and DENV2 NS5-RRV (Tetravalent) (Virus-vectored vaccine)	Dengue virus 2 E85 and NS5 protein expressed from RR virus vector	Preclinical/NHPs	(51)
DENV1 C/prM/E (VLPs vaccine)	Co-expressed dengue virus 1 C, prM and E protein in *P. pastoris*	Preclinical/Rabbit	(81)
DENV2 EDIII-HBsAg or ectoE (Tetravalent) (VLPs vaccine)	Dengue virus 2 EDIII protein-HBsAg VLPs or ectoE-based VLPs expressed in *P. pastoris*	Preclinical/Mice	(82)
DENV1 E; DENV2 E; DENV3 E, and DENV4 E (Monovalent) (VLPs vaccine)	Dengue virus 1, 2, 3 and 4 E ectodomain expressed in *P. pastoris*	Preclinical/Mice	(83–86)
DENV1-4 prM/E (Tetravalent) (VLPs vaccine)	Co-expressed dengue virus 1-4 prM and E protein in *P. pastoris*	Preclinical	(87)
DENV1-4 prM/E (VLPs vaccine)	Co-expressed dengue virus 1-4 prM and E protein with a F108A mutation in the fusion loop of E in *P. pastoris*	Preclinical/Mice	(88)
DENV1-2/mVLP (Bivalent) (VLPs vaccine)	Co-expressed dengue virus 1 and 2 E protein mosaic VLP (mVLP) in *P. pastoris*	Preclinical/Mice	(89)
DSV4 (Tetravalent) (VLPs vaccine)	Co-expressed dengue virus 1-4 EDIII protein + HBsAg (4 copies) in *P. pastoris*	Preclinical/mice/NHPs	(90)
T-mVLPs (Tetrvalent) (VLPs vaccine)	Dengue virus 1-4 E protein expressed in *P. pastoris*	Preclinical/Mice	(91)
MWNT-DENV3 E (Recombinant protein vaccine)	Dengue virus 3 E protein expressed in *E. coli* and conjugated with MWNT	Preclinical/ mice	(92)
DENV4 EDIII (Recombinant protein vaccine)	Dengue virus 4 EDIII protein expressed in *E. coli*	Preclinical/Mice	(93)
DENV1-4 EDIII (Recombinant protein vaccine)	Dengue virus 1-4 EDIII proteins expressed in *E. coli*	Preclinical/Mice	(15)
DENV2 EDIII (Recombinant protein vaccine)	Dengue virus 2 EDIII protein expressed in *E. coli*	Preclinical/Mice	(94)
DENV1 EDIII (Recombinant protein vaccine)	Dengue virus 1 EDIII protein expressed in *E. coli*	Preclinical/Mice	(95)
DENV3 EDIII (Recombinant protein vaccine)	Dengue virus 3 EDIII protein expressed in *E. coli*	Preclinical/Mice	(96)
Lipidated DENV3 EDIII and DENV4 EDIII (Recombinant protein vaccine)	Lipidated dengue virus 3 and 4 EDIII proteins expressed in *E. coli*	Preclinical/Mice	(97, 98)
DENV cEDIII (Tetravalent) (Recombinant protein vaccine)	Dengue virus consensus EDIII protein expressed in *E. coli*	Preclinical/NHPs	(99)
Lipidated DENV1-4 EDIII (Tetravalent) (Recombinant protein vaccine)	Physical mix of lipidated EDIII proteins of dengue virus 1-4 expressed in *E. coli*	Preclinical/Mice	(100)
DENV2 EDIII-C (Recombinant protein vaccine)	Dengue virus 2 EDIII-capsid chimeric protein expressed in *E. coli*	Preclinical/Mice	(101)
DENV4 EDIII-P64k and DENV EDIII-P64k (Tetravalent) (Recombinant protein vaccine)	Dengue virus 4 EDIII-P64k chimeric protein and tetravalent EDIII-P64k expressed in *E. coli*	Preclinical/Mice	(102)
EDIII 1, 2 I-P64k and EDIII-capsid (Monovalent) (Recombinant protein vaccine)	EDIII-P64k (chimeric) and EDIII capsid (chimeric) proteins expressed in *E*. *coli*	Preclinical/NHPs	(103–105)
DENV1-4 C (Recombinant protein vaccine)	Dengue virus 1-4 capsid proteins expressed in *E. coli*	Preclinical/NHPs	(106)
DIIIC and Tetra DIIIC (Tetravalent) (Recombinant protein vaccine)	Dengue virus 1-4 DIII capsid chimeric proteins of each dengue virus serotypes expressed in *E. coli* and their mixture	Preclinical/NHPs	(107, 108)
MixBiEDIII (Recombinant protein vaccine)	Mix of EDIIIs proteins of two serotypes (1–2 and 3–4) expressed in *E. coli*.	Preclinical/Mice	(109)
EIII-STF2 (Tetravalent) (Recombinant protein vaccine)	Tetravalent dengue vaccines consisting of four constructs, each containing two copies of EIII fused to flagellin (STF2) and expressed in *E. coli*	Preclinical/NHPs	(110)
DENV3 EDIII (Recombinant protein vaccine)	Dengue virus 3 EDIII protein expressed in *E. coli*	Preclinical/Mice	(111)
DENV1 EDIII and DENV2 EDIII (Recombinant protein vaccine)	Dengue virus 1 and 2 EDIII proteins expressed in *E. coli*	Preclinical/Mice	(112)
DENV2 NS1 (Recombinant protein vaccine)	Dengue virus 2 NS1 protein expressed in *E. coli*	Preclinical/Mice	(113, 114)
DENV2 NS3 (Recombinant protein vaccine)	Dengue virus 2 NS3 protein expressed in *E. coli*	Preclinical/Mice	(115)
DENV2 NS5 (Recombinant protein vaccine)	Dengue virus 2 NS5 protein expressed in *E. coli*	Preclinical/Mice	(116)
DENV2 E (Recombinant protein vaccine)	Dengue virus 2 E protein expressed in *P. pastoris*	Preclinical/Mice	(117)
DENV1-4 EDIII (Tetravalent) (Recombinant protein vaccine)	Dengue virus 1-4 EDIII chimeric protein expressed in *P. pastoris*	Preclinical/Mice	(118)
DENV2 EDIII (Recombinant protein vaccine)	Dengue virus 2 EDIII protein expressed in *P. pastoris*	Preclinical/Mice	(119)
DENV1-4 cEDIII (Recombinant protein vaccine)	Dengue virus 1-4 consensus EDIII protein expressed in *S.cerevisiae*	Preclinical/Mice	(120)
V180 (DEN-80E), Merck/NIAID (Recombinant protein vaccine)	Truncated (80% E protein) expressed in S-2 cells	Phase I	(121)
DENV 1-4 EDIII (Recombinant protein vaccine)	Dengue virus 1-4 EDIII proteins expressed in *Trichoplusia ni* cells (High Five cells)	Preclinical/ Mice	(122)
DENV2 EII*EIII and DENV2 EIII*EIII/NS1 (Recombinant protein vaccine)	Dengue virus 2 EII*EIII and EIII*EIII/NS1 proteins expressed in S-2 cells	Preclinical/ Mice	(123)
DENV2 NS1 (Recombinant protein vaccine)	Dengue virus 2 NS1 protein expressed in S-2 cells	Preclinical/Mice	(124)
DENV1 E, DENV2 E and DENV 4 E (Recombinant protein vaccine)	Dengue virus 1, 2 and 4 E proteins expressed in Sf-9 cells	Preclinical/ Mice	(125)
DEN-80E LNPs (Tetravalent), Merck (Recombinant protein vaccine)	Dengue virus E protein from all four serotypes (DEN-80E) expressed in S-2 cells formulated with ionizable cationic lipid nanoparticles (LNPs)	Preclinical/NHPs	(126)
cE80(D4) and cE80(max)] (Recombinant protein vaccine)	Dengue proteins single and consensus [cE80(D4) and cE80(max)] expressed in Sf−9 cells	Preclinical/Mice	(127)
DENV1-4 cEDIII (Recombinant protein vaccine)	Dengue virus consensus EDIII protein in conjunction with PIGS expressed in CHO cells	Preclinical/Mice	(128)
DENV2 EDIII (Recombinant protein vaccine)	Dengue virus 2 EDIII protein expressed using TMV based vector in *N.benthamiana*	Preclinical/Mice	(129)
DENV1-4 cEDIII (Recombinant protein vaccine)	Dengue virus consensus EDIII protein in conjunction with PIGS expressed in *N. benthamiana*	Preclinical/Mice	(128)

An NS1 based DNA vaccine candidate (pcTPANS1) encoding the t-PA signal sequence linked to NS1 gene of dengue virus 2, elicited high antibody response and further provided protection in 97% mice from challenge with dengue virus 2, without producing neutralizing antibodies (70, 130). Two vaccine candidates (αDEC-NS1 and αDCIR2-NS1) containing NS1 gene of dengue virus 2 targeting different dendritic cell population were transfected in HEK cells and their immune responses were studied in BALB/c mice. Further, αDEC-NS1 immunized mice were protected upon a lethal intracranial challenge with the dengue virus 2 compared to mice immunized with αDCIR2-NS1 (71). Another plasmid DNA vaccine encoding prM and E proteins of dengue virus 3 was utilized to immunize mice which produced neutralizing antibodies that protected mice against lethal challenge (72). Evaluation of another NS1 based DNA vaccine loaded on PLGA/PEG microspheres in BALB/c mice resulted in the greatest survival of mice upon challenge study (131). In yet another approach, a DNA vaccine construct containing prM and E genes of dengue virus 4 was generated using a mammalian expression plasmid (pCI). This DNA plasmid vaccine protected mice from dengue virus 4 infection (73). The various factors responsible for expression, secretion and folding of E ectodomain from dengue virus 1-4 were studied in mammalian cells which confirmed the role of proper folding of DII for efficient E ectodomain secretion. Thus, the proper DII folding clearly affected neutralizing antibody responses in DNA vaccinated mice, which will lead to the development of E-based DNA vaccines in future (17). A plasmid DNA vaccine candidate encoding EDIII protein of dengue virus linked to CH3 domain of the IgG immunoglobulin H chain was constructed. This plasmid DNA vaccine was able to produce neutralizing antibody titers against dengue virus 1-4. Immunization with a tetravalent formulation in mice demonstrated neutralizing antibodies against all four dengue virus 1-4 (74). Recently, a novel construct has been designed using a multimeric scaffold of *Geobacillus stearothermophilus* E2 proteins presenting EDIII protein of dengue virus 2 and a DNA expression plasmid encoding for EDIII gene. Rhesus macaques were immunized using simultaneous intramuscular injection of protein and plasmid DNA delivered using gene gun. This vaccine conferred protection to the vaccinated macaques upon challenge with dengue virus 2, whereas the control animals showed viremia. These results concluded that a neutralizing antibody titer (>1: 6000) provided sterilizing protection in immunized macaques (75).

## Virus-vectored dengue vaccines

The list of some virus-vectored based dengue vaccine candidates is given in Table 2. The virus-vectored vaccine uses viruses as vector for expressing antigens of dengue virus *in vivo*. Some of the viruses used as vector are Adenovirus, Alphavirus, and Vaccinia virus (10, 17, 67, 68). Measles virus (MV) vector is safe and functions like a biological adjuvant. A virus-vectored dengue vaccine candidate expressing a single tetravalent dengue virus antigen was designed. This construct contains the EDIII of dengue virus 1–4 as well as M protein ectodomain (ectoM) of dengue virus 1. A live attenuated MV vaccine vector was employed to express MV-DEN chimera. This chimeric protein induced neutralizing antibodies and further activated cellular immunity in mice. The ectoM protein in the vaccine construct provided adjuvant effect. This MV based tetravalent vaccine produced neutralizing antibodies in mice and NHP against dengue virus 1-4 (66, 76). A virus-vectored vaccine candidate based on Alphavirus, namely Venezuelan equine encephalitis (VEE) virus replicon particles (VRP) were developed. These VRPs expressing soluble E dimers [E85] from dengue virus 1-4 were immunized in macaques in 2 doses and subsequently protected them against dengue virus. This study showed that a tetravalent E85-VRP dengue vaccine elicited a simultaneous and protective response to dengue virus 1-4. This tetravalent vaccine provided balanced response and protection in macaques thereby putting forth this candidate for human trials (77). Further a single dose of tetravalent VRP vaccine was able to induce both neutralizing antibody and T-cell responses to each serotype in neonatal and adult BALB/c mice. However, the neonatal mice immune response was lower in magnitude than the response in adult BALB/c mice (132). In yet another study, two MV vectors were designed to express dengue virus type 2 EDIII protein and surface antigen (S) of Hepatitis B virus. The first construct produced only EDIII-S whereas second one produced hybrid antigen displaying DIII-S on an unmodified HBsAg scaffold. This study showed that the second MV vector was immunogenic in MV-susceptible mice and produced strong neutralizing responses against dengue virus 2. However, the first MV vector produced immunity against measles virus alone (78). A tetravalent dengue vaccine candidate based on a recombinant MV vector expressing EDIII of dengue virus 1 to 4 was constructed and immunized in mice. This vaccine candidate elicited neutralizing antibody response against both MV and dengue virus 1-4. This vaccine candidate also conferred protection to the mice against all the serotypes of dengue virus (79).

A recombinant modified vaccinia virus Ankara (rMVA) having an intact ER (endoplasmic reticulum) signal peptide based dengue vaccine containing dengue virus 3 E protein (rMVA/Sg-E) elicited neutralizing antibody titers which protected the C57BL/6 mice upon intracranial challenge with homologous virus (80). Recently, Rhesus monkey Rhadinovirus (RRV) was employed to construct two vaccine candidates. The first candidate expressed dengue virus 2 NS5 protein whereas a second one expressed soluble E protein (E85) of dengue virus 2. Four macaques were immunized with a single dose of recombinant RRVs and were subsequently challenged with dengue virus 2. This candidate induced neutralizing antibodies against dengue virus 2 in all four vaccinated monkeys. Further study is warranted to evaluate the efficacy of Herpes virus and other persisting virus vectors toward the development of a successful dengue vaccine candidate (51).

## Virus-like particles (VLPs) based dengue vaccines

The list of VLPs based dengue vaccine candidates is given in Table 2. The VLP vaccines lack replicative genetic material but present antigen in the same manner as mature dengue virion. The co-expression of C, prM and E proteins of dengue virus 1 was carried out in *P. pastoris* which resulted in the spontaneous formation of VLPs. Furthermore, these VLPs demonstrated immunogenicity in rabbit (81). A chimeric protein containing EDIII of dengue virus 2 and core antigen of Hepatitis B virus was expressed as VLPs in *P. pastoris* and has been found to be immunogenic (82). *P. pastoris* expressed dengue virus 2 E ectodomain, was found to form VLPs, in the absence of prM, which is responsible for inducing ADE phenomenon. These VLPs produced type-specific neutralizing antibodies that protected mice from lethal dengue virus 2 (83). In another approach, four dengue virus-VLP serotypes were expressed in *P. pastoris*, based on co-expression of the prM and E proteins. The protection rates of tetravalent dengue virus -VLP immune sera against challenge with dengue virus 1-4 serotypes in suckling mice were 77, 92, 100, and 100%, respectively, indicating greater protective efficacy compared with monovalent immune sera. These results provide an important basis for the development of VLP based dengue vaccine (87). Similar to dengue virus 2 ectodomain (83), dengue virus 1, 3, and 4 VLPs were also produced in *P. pastoris* that elicited antibodies response as well as type specific neutralizing antibodies in mice (84–86).

A novel tetravalent dengue vaccine based on VLPs approach was also designed by co-expressing prM and E proteins from dengue virus 1-4 with a F108A mutation in the fusion loop of E protein. This mutation led to the increased production of VLPs in HEK cells. The optimized dengue virus 1-4 VLPs were purified by a combination of an anion exchange and hydroxyapatite chromatography. These vaccine constructs demonstrated high levels of neutralization activity in mice immunized with individual, monovalent and tetravalent dengue virus 1-4 VLPs. The neutralization potential of VLPs was significantly higher than that of DNA or recombinant E proteins. Moreover, ADE effect was not found against any of the four dengue virus serotypes even at a serum dilution of 1:10 (88).

Recently, a bivalent mosaic VLP (mVLP) was produced by co-expressing the E protein of Dengue virus 1 and 2 in *P. pastoris*. This mVLP elicited EDIII specific virus neutralizing antibodies against Dengue virus 1 and 2 in BALB/c mice. The ADE potential of this mVLP was evaluated in AG129 mice which showed that these VLPs did not induce ADE inducing antibodies (89). Further, a tetravalent VLP (DSV4) was designed by fusion of EDIII of all the four serotypes and HBsAg, which was co-expressed with unfused S antigen to form mVLP. The DSV4 VLPs were highly immunogenic and induced potent and durable neutralizing antibodies against all the four serotypes in mice and macaques. These murine antibodies protected the mice against challenge with dengue virus 4 (90). Another recent approach involved use of a plasmid capable of expressing all four E genes of dengue virus in *P. pastoris*. This tetravalent mosaic VLPs (T-mVLPs) elicited EDIII-directed antibodies in mice which could neutralize all four dengue virus serotypes (91). These works on VLPs demonstrated that the E based tetravalent dengue vaccine candidate can be a potential subunit vaccine.

## Recombinant subunit proteins based dengue vaccines

The selection of recombinant protein expression systems depends on major factors namely protein yield, its quality, ability to scale-up, time and cost of production. Most of the recombinant dengue virus proteins for vaccine development studies are produced in either bacteria (*E. coli*), yeast (*S. cerevisiae* and *P. pastoris*), mammalian cells (HeLa, HEK, Vero and BHK), insect cells (Sf-9 and S-2), or transgenic plants (*Nicotiana tabacum* and *N. benthamiana*) (17, 20, 74, 86, 112, 133, 134). Recombinant microbial production systems have already delivered a successful vaccine against Hepatitis B with proven safety record. The recently licensed Dengvaxia is unable to provide complete protection in humans and has led to increased hospitalization rates in seronegative humans due to disease enhancing antibodies (8). Therefore it has raised serious concern regarding the safety of live attenuated vaccines. Thus, alternative dengue vaccines are the need of time based on other approaches. In dengue vaccine development, an alternative approach to live attenuated/purified inactivated vaccines is the recombinant protein based dengue subunit vaccines. The main advantage of a recombinant subunit vaccine for dengue includes balanced immune response against all four dengue virus serotypes. Further, it was also reported that the recombinant subunit vaccine needed a low antigen dose for vaccination (11, 135). It also reduces the risk of ADE (89). In addition, recombinant vaccines are believed to offer full protection in a significantly shorter time-frame than that needed for live attenuated vaccines. The dengue virus E protein or its DIII and NS1 protein have been the main focus of recombinant subunit vaccine development using various approaches like the fusion with other components or use of adjuvants and many others (20).

## Recombinant subunit protein based dengue vaccine candidates produced in *E. coli*

Various researchers studied the vaccine potential of *E. coli* expressed recombinant dengue virus proteins. However, *E. coli* has also some disadvantages such as lack of PTM, improper protein refolding and endotoxin issue (12, 24). The lists of some recombinant dengue virus proteins produced in *E. coli* for vaccine development are given in Table 2. In a novel approach, a multi-walled carbon nanotubes (MWNT) conjugated with dengue virus 3 E protein (MWNT-DENV3E) was developed. The immunogenicity of MWNT-DENV3E was evaluated in BALB/c mice and generated higher titers of neutralizing antibodies (92).

Immunomodulatory potential of refolded dengue virus 4 EDIII protein in combination with various adjuvants [Freunds Complete adjuvant (FCA), Montanide ISA720, Alum] revealed that the FCA formulation resulted in high antibody titers in mice that blocked the virus entry *in vitro* (93). A metal affinity membrane chromatography purified recombinant EDIII antigens from dengue virus serotypes 1–4 were successfully tested for their ability to induce EDIII specific antibodies in mice. These antibodies successfully neutralized the respective serotypes of the dengue virus (15). Affinity and cation-exchange chromatography purified EDIII protein of the dengue virus 2 elicited high titers of neutralizing antibodies in mice. Further, these antibodies protected suckling mice from virus challenge (94). Dengue virus 1 EDIII protein along with PELC and CpG Oligodeoxynucleotides (ODN) produced neutralizing antibodies against dengue virus 1. Upon *in vitro* re-stimulation of splenocyte, an increased IFN-γ level was obtained (95). In another strategy, a recombinant lipidated dengue virus 1 EDIII (LD1EDIII) and its non-lipidated form, dengue virus 1 EDIII, with an adjuvant (PELC) enhanced the immunogenicity when compared to either combining dengue virus 1 EDIII with PELC or the antigen alone (136). Another different approach was used for designing a single tetravalent vaccine candidate. For this purpose, a consensus EDIII protein among all the four dengue virus serotypes was produced in *E. coli*. This tetravalent antigen in combination with alum adjuvant elicited high levels of neutralizing antibody against all the four serotypes of dengue virus in mice. When this candidate was evaluated in NHPs, two out of three monkeys produced neutralizing antibody titers against dengue virus 2, but failed to produce neutralizing antibodies against dengue virus 1, 3, and 4. In addition to this, a recombinant lipidated EDIII (as monovalent form) and a lipidated consensus EDIII were also developed. This lipidated approach eliminated the requirement of adjuvant. In case of lipidated EDIII of dengue virus 4, the neutralizing antibody titers were significantly higher in comparison to that of non-lipidated EDIII. The lipidated EDIII also reduced viraemia significantly upon challenge with dengue virus 4 (97, 99, 136). In another approach, recombinant dengue virus 3 and 4 EDIII proteins were expressed in lipidated form and found to induce neutralizing antibodies capable of reducing viremia level upon virus challenge. However, alum along with dengue virus 3 EDIII protein could not induce neutralizing antibody response and further reduced viraemia upon challenge (97, 98). Physical mixture of four individual recombinant lipidated dengue EDIII proteins also induced both humoral and cellular immune response against all four serotypes of dengue virus in mice. This study utilized the intrinsic adjuvant properties of recombinant lipoproteins so as to preclude the use of adjuvant (100). Scaled up recombinant EDIII proteins of dengue virus 1 and 2 were also studied for their immunogenicity in mice and shown to elicit high antibody titers (112).

A chimeric dengue vaccine containing two copies of dengue virus 4 EDIII gene together with P64k gene (carrier protein of *Neisseria meningitides*) was successfully expressed. This protein induced partial protection in mice (102). Further, recombinant dengue virus 1 and 2 EDIII fusion proteins (P64k) elicited high titers of neutralizing antibodies in monkeys against different strains of dengue virus 1 and 2 (103). A DIII-C chimeric protein of dengue virus 2 was expressed using *E. coli* and induced a functional immune response and provided protection in mice (101). In yet another approach, a tetravalent formulation combining the DIII-P64k recombinant proteins of dengue virus 1, 3, and 4 with the DIII-C protein from dengue virus 2 were prepared. Thus, a tetravalent formulation containing DIII of the E protein fused to the C protein was able to induce neutralizing antibodies against dengue virus 1, 2, and 3 along with high level expression of IFN-γ thereby suggesting it as a potential vaccine candidate (137). Evaluation of a tetravalent vaccine formulation (EDIII-P64K) adjuvanted with alum was also carried out in mice for its immunogenicity. After three immunizations with this formulation, the high titers of neutralizing antibodies were observed against all the four serotypes of dengue virus. However, the seroconversion rate against dengue virus 4 was found to be lowest. These animals were subjected to virus challenge after 1 month which resulted in partial but statistically significant protection (138). The chimeric protein consisting of EDIII and C protein (EDIII-C) of the dengue virus 1-4 was expressed in *E. coli* for future vaccine studies (139). Further, monovalent and tetravalent formulations were studied in NHPs and elicited neutralizing antibody titers (107). *E. coli* expressed dengue virus 2 EDIII-C fusion protein (DIIIC-2) was also adjuvanted with ODNs to enhance the immunogenicity. Recently, it was demonstrated that this protein when delivered along with alum in monkeys resulted in activating both arms of immune response-humoral and cellular immunity. Further, two of three immunized monkeys were completely protected against challenge (104, 139). Another tetravalent vaccine candidate based on *E. coli* expressed and ion exchange purified recombinant dengue virus C protein was also studied. This tetravalent antigen when adjuvanted with ODN containing immunostimulatory CpG motifs induced an IFN-γ response (a cytotoxic T-cell response) in mice and monkeys. This led to reduced viraemia upon challenge with dengue virus without producing virus specific antibodies. Therefore, this new vaccine candidate may not carry the risk for disease enhancement associated with antibodies elicited by other vaccine formulations (106). A physical tetravalent mixture containing DIII and C protein of each dengue virus serotype (Tetra DIIIC) was evaluated in monkeys. This protein boosted neutralizing antibody responses previously generated in monkeys. These results suggested the potential use of a Tetra DIIIC as a dengue vaccine candidate (108).

Two BiEDIII constructs, one containing EDIIIs of type 1 and 2, and the second containing EDIIIs of type 3 and 4 in tandem using the linkers [(Gly4Ser)3] were designed. Both the bivalent recombinant EDIIIs proteins were expressed in *E. coli*, separately and mixed in equal proportion to form a tetravalent vaccine candidate. This tetravalent antigen was used for immunization of BALB/c mice. Furthermore, in the suckling mouse model, immune sera of mice conferred protection against challenge with dengue virus 1-4 (109). In yet another approach, four tetravalent constructs were designed each containing two copies of EIII linked with bacterial flagellin protein, a Toll-like Receptors (TLR) 5 ligand (R3.2x format) which induced solid and long termed neutralizing antibodies in rhesus macaques. This study demonstrated that, flagellin-EIII fusion based vaccines are immunogenic and protected NHPs model partially (110). Recently, a novel resurfaced immunogen (rsDIII) has been designed by mutating non-neutralizing epitopes on it. This rsDIII elicited broadly neutralizing antibodies against dengue virus 1-3 in mice but failed to protect AG129 mice against challenge (140).

A purified recombinant NS1 protein and a nontoxic lethal toxin (LT) derivative is a promising alternative for the generation of safe and effective protein-based dengue vaccine (113). Further, modification of NS1 proteins by deleting their cross-reactive epitopes could lead to development of a safer and effective vaccine against dengue virus infection (141).

The dengue virus NS3 protein is also one of the important targets for T-cell response during infection. The diagnostic potential as well as immunogenicity of a recombinant NS3 protein of dengue virus 1-4 was studied (142). Another study on the antigenicity and immunogenicity of *E. coli* expressed dengue virus 2 NS3 protein was carried out. This two-step purified protein was able to stimulate a Th-1-type response in mice which suggests that it may be incorporated in future dengue vaccine candidates (143). In a recent report, it was concluded that a purified recombinant NS3 helicase protein may be included in the dengue virus 2 inactivated vaccines. It can synergistically elicit a more effective immune response (115). Recently, purified NS5 protein partially protected mice from lethal challenge with the dengue virus type 2 NGC strain and with a clinical isolate. This study suggested that it is a potential vaccine candidate which can elicit protective immune response (116).

## Recombinant dengue virus subunit protein vaccines produced in yeast

The list of some recombinant dengue virus proteins produced in yeast is given in Table 2. Majority of VLPs based dengue vaccine candidates were produced using *P. pastoris* as discussed in earlier section (Table 2). The truncated E protein from dengue virus 2 was expressed in *P. pastoris* which elicited anti-dengue antibodies in mice (117). In another approach, an EDIII based chimeric protein of dengue virus expressed in *P. pastoris* could induce neutralizing antibodies against dengue virus 1-4 (118). Similarly, dengue virus type 2 EDIII protein also elicited virus-neutralizing antibodies (119). A synthetic consensus gene of dengue virus EDIII (scEDIII) from all four serotypes was expressed using *S. cerevisiae*. Upon immunization with this purified scEDIII protein in mice, the antibodies raised against this protein was found to be specific to dengue virus 1-4. It also resulted in balanced immune response against dengue virus 1-4. Thus, it was inferred that a tetravalent dengue vaccine can be formulated (120). A fusion construct (Tet-EDIII-Co1) that comprises of Co1 (an M cell-specific peptide ligand) at the C-terminus of a recombinant tetravalent EDIII gene (Tet-EDIII) from dengue virus 1-4 was also expressed in *S. cerevisiae*. Further evaluation of binding ability of this construct may lead to the development of an oral dengue vaccine (144).

## Recombinant dengue virus subunit protein vaccines produced in insect cells

The list of some recombinant dengue virus proteins produced in insect cells is given in Table 2. The most promising recombinant subunit vaccine candidate is V180 (Merck). This vaccine candidate consists of a recombinant truncated protein containing 80% of the N-terminal dengue virus E protein (DEN-80E). These proteins were efficiently produced using the Schneider-2 (S-2) Drosophila cell line. For tetravalent vaccine production, this protein has been expressed in the S-2 expression system for each of the four dengue virus serotypes. A mixture of all four 80E recombinant subunits and ISCOMATRIX adjuvant was used for immunization in mice. These immune sera resulted in effective virus neutralization of dengue virus 1-4. The neutralizing antibody response to the tetravalent mixture components was similar to each of individually administered recombinant subunits response. The 80E subunit of dengue virus 2 was also evaluated using mice and monkey. Further, in the vaccine formulation with low doses of protein, it provided protection in mice and monkey against virus challenge. This report established the potential of S-2 cell produced 80E subunits based tetravalent dengue vaccine (145). Further, preclinical development of this candidate demonstrated the induction of strong neutralizing antibody titers after virus challenge and resulted in protection against viremia (even a gap of 8–12 months after last vaccine dose) (135). This candidate has completed phase I trial (10, 121, 135). Another novel approach involved a tetravalent antigen comprising of DEN-80E from dengue virus 1-4 serotypes adjuvanted with lipid nanoparticles (LNPs), was used for immunization in rodents (mice, Guinea pigs), and NHPs (Rhesus macaques). This study confirmed that the LNPs elicited high neutralizing antibody titers against dengue virus thereby ruling out the strategy of using TLR agonist, unlike the case of HIV (146). This vaccine formulation was well-tolerated and safe (126).

In another study, the E genes from dengue virus 1, 3, and 4 (three isolates of dengue virus namely) were expressed using Sf-9 cells. Recombinant E proteins also induced cellular immune responses in immunized mice, as revealed by secretion of interleukin 3 (IL-3) (125). A single consensus E80 [cE80(max)] among 3127 dengue virus strains based recombinant protein was expressed in Sf-9 cells. This vaccine candidate elicited neutralizing antibody responses to dengue virus serotypes 1-4. The cE80(max) elicited IgG1 subtype antibody. Further, this vaccine activated virus-specific Th2 response and weak Th1 responses that secreted IL-4 and IFN-γ respectively. This report suggested that the future studies are required to improve this vaccine candidate for eliciting stronger and more balanced antibody responses (127). Recombinant dengue virus EDIII proteins were also produced using High Five cells and immunized in mice individually, and in tetravalent combination. They produced serotype-specific IgG1 neutralizing antibodies (122).

A fusion proteins (i.e., rEII^*^EIII and rEII^*^EIII/NS1^*^) of dengue virus 2 was expressed in S-2 cell system either alone or fused to 3 copies of P28 (the minimum CR2-binding domain of the complement protein C3d). The immunogenicity of these four proteins in BALB/c mice resulted in induction of specific antibody response. The immune response was greater with the P28 fusion proteins in the mice. All these proteins produced high titers of neutralizing antibodies in BALB/c mice (123). Recombinant dengue virus 2 NS1 protein produced using S-2 cells was immunized in *Ifnar*^−/−^ mice. Vaccination with dengue virus 2 NS1 protein conferred protection against lethal challenge with homologous virus. Moreover, NS1 vaccination from dengue virus 1, 3, 4 provided significant protection against dengue virus 2 challenge (124).

## Recombinant dengue virus subunit protein vaccines produced in mammalian cells

The list of some dengue virus recombinant proteins produced in mammalian cells is given in Table 2. Some of the DNA based dengue vaccine candidates have been transfected using mammalian cells and some of the VLPs based dengue vaccine candidates produced using mammalian cells are already described in earlier sections. In a novel approach, a polymeric immunoglobulin G scaffold (PIGS) was constructed in order to increase the uptake of vaccine candidates by the immune cells *in vivo*. A consensus EDIII sequence of dengue virus was linked to PIGS and produced in CHO cells as well as in transgenic plants (*N. benthamiana*). These proteins induced a high titered IgG antibody response in mice with or without adjuvant which showed a neutralizing potential against the dengue virus 2 (128).

## Recombinant dengue virus subunit protein vaccines produced in transgenic plants

The list of some dengue virus recombinant proteins produced in transgenic plants is given in Table 2. Dengue virus 2 EDIII protein was produced in *N. benthamiana* using a Tomato mosaic virus (TMV) based vector system. This expressed protein was immunogenic in mice and produced neutralizing antibodies. This report provided the first evidence of a potential plant-based dengue recombinant subunit vaccine (129). Production of recombinant EDIII protein of dengue virus 2 was also carried out in non-nicotine transgenic tobacco plants and demonstrated the suitability of plant-based system for development of a dengue vaccine candidates (147). However, this study did not used any animal model to determine vaccine potential of this protein.

In another approach, a B subunit of *Vibrio cholera* toxin (CTB) subunit and consensus EDIII protein of dengue virus (cEDIII-CTB) was also expressed in transgenic rice callus. This cEDIII-CTB also showed the potential of the plant based ligand fusion proteins as oral dengue vaccine since it activates the mucosal immune system to improve the overall immune responses (148). Various other dengue virus proteins were also expressed using plant systems but these proteins were not evaluated in mice or any other models for immunization (11). As mentioned earlier, the consensus DIII sequence (cEDIII) of dengue virus in conjunction with PIGS was expressed in tobacco plants (*N. benthamiana*) also, apart from CHO cells. This study established its immunogenicity and induced neutralizing antibody in mice (128). Recently, subunit dengue vaccine based on the consensus EDIII domain and human IgG1 polymeric scaffold have been produced in *N. benthamiana*. This humanized D-PIGS induced both humoral and cellular immune responses, that are essential for protection. However, this candidate needs further evaluation in NHPs (149).

## Conclusions and future perspectives

The global burden of dengue is rapidly increasing and will continue till an effective vaccine against dengue is developed. Various vaccine candidates for dengue are at preclinical and clinical trials stages of development based on different approaches. These vaccines include tetravalent, live attenuated virus, purified inactivated virus, recombinant proteins subunit, plasmid DNA, and virus-vectored dengue vaccines. A comprehensive evaluation of humoral and cell mediated immune response is critical for successful dengue vaccine development. A fully efficacious dengue vaccine candidate is still a challenge even after several decades of research on dengue. The only dengue vaccine candidate, Dengvaxia which has been licensed recently was unable to deliver a balanced immune response against dengue virus serotypes 1-4. Thus, the development of a fully efficacious dengue vaccine is the need of time. This vaccine should provide long lasting protection against all the serotypes of dengue virus in a balanced manner and should also overcome ADE effect. Recent progress on recombinant subunit based dengue vaccine candidates using different type of approaches such as fusion with other immunogenic epitopes, use of novel adjuvant, protein expression in different host systems, designer vaccine, and VLPs development will open new avenues for the development of fully efficacious vaccine against dengue.

The recombinant proteins based dengue subunit vaccines are gaining importance these days due to their safety features, easy scale-up, economical, and balanced tetravalent immune response. It also obviates the inherent risk of ADE phenomena associated with chimeric live attenuated vaccine. The dengue vaccine candidates in preclinical stages cover various types of approaches in terms of antigen and its delivery. Carefully designed studies in NHPs/novel humanized mice models should allow prioritization of preclinical candidates for human trials. With many dengue vaccine candidates in various stages of clinical trials, the coming decade will see more numbers of the licensed dengue vaccine based on recombinant proteins approach.

## Author contributions

All authors listed have made a substantial, direct and intellectual contribution to the work, and approved it for publication.

### Conflict of interest statement

The authors declare that the research was conducted in the absence of any commercial or financial relationships that could be construed as a potential conflict of interest.
